# Lessons from the Endoplasmic Reticulum Ca^2+^ Transporters—A Cancer Connection

**DOI:** 10.3390/cells9061536

**Published:** 2020-06-24

**Authors:** Xingjian Zhai, Andra Mihaela Sterea, Yassine El Hiani

**Affiliations:** 1 Department of Physiology and Biophysics, Faculty of Medicine, Dalhousie University, Halifax, NS B3H 4R2, Canada; xn980835@dal.ca; 2 Faculty of Dentistry, Dalhousie University, Halifax, NS B3H 4R2, Canada; andra.sterea@dal.ca

**Keywords:** endoplasmic reticulum, calcium, IP_3_R, RyR, SERCA, STIM/Orai

## Abstract

Ca^2+^ is an integral mediator of intracellular signaling, impacting almost every aspect of cellular life. The Ca^2+^-conducting transporters located on the endoplasmic reticulum (ER) membrane shoulder the responsibility of constructing the global Ca^2+^ signaling landscape. These transporters gate the ER Ca^2+^ release and uptake, sculpt signaling duration and intensity, and compose the Ca^2+^ signaling rhythm to accommodate a plethora of biological activities. In this review, we explore the mechanisms of activation and functional regulation of ER Ca^2+^ transporters in the establishment of Ca^2+^ homeostasis. We also contextualize the aberrant alterations of these transporters in carcinogenesis, presenting Ca^2+^-based therapeutic interventions as a means to tackle malignancies.

## 1. Introduction

First revealed via light microscopy by French cytologist Garnier in 1897, the endoplasmic reticulum (ER) quickly became the topic of interest for many research endeavors [1]. Sharing a partial intersection with the nucleus and stretching to the cellular periphery, the ER consists of convoluted networks of cisternae that serve as specialized sites to facilitate the translation, modification, folding, sorting, and trafficking of proteins [2]. Perhaps the most important function of the ER, however, is its capacity to operate as the major Ca^2+^ storage organelle responsible for the maintenance of global Ca^2+^ homeostasis [3]. It has been long known that in order for eukaryotic cells to coordinate complex cellular events, they must employ specialized signaling molecules that warrant the transmission of extracellular signals into intracellular responses. Among these signaling molecules, Ca^2+^ represents the evolutionary choice of living cells [4]. Indeed, Ca^2+^ has been a conserved second messenger from the early days of prokaryotic existence and has evolved to virtually cover all essential functions in a cell [5]. This strategic positioning of Ca^2+^ within the eukaryotic signal transduction network is mainly due to its unique polarizability and coordination chemistry that accommodate reversible binding with Ca^2+^-sensing proteins [6]. The degree of subtlety encoded in the intensity, duration, amplitude, and downstream effector landscape of Ca^2+^ signals makes it one of the most versatile molecules supporting eukaryotic life [7]. Given the pivotal role of Ca^2+^ in maintaining cellular physiology, it is unsurprising that the systematic regulation of intracellular Ca^2+^ has become a hotspot for manipulation by various human pathologies, including cancer, a multifactorial disease seamlessly exemplifying such paradigm [8]. In their seminal review, Hanahan and Weinberg proposed the “hallmarks of cancer” in an effort to illustrate the defining features and mechanisms associated with oncogenic states [9]. Importantly, most of these hallmarks stem from alterations in the ER Ca^2+^ signaling milieu as they have been characterized in a broad range of clinical malignancies [10]. Here, we will examine the mechanisms and critical roles of homeostatic handling of ER Ca^2+^ by ER Ca^2+^ transporters and their dysregulations in cancer pathogenesis.

## 2. ER Topography and Ca^2+^ Handling

**Figure 1:**
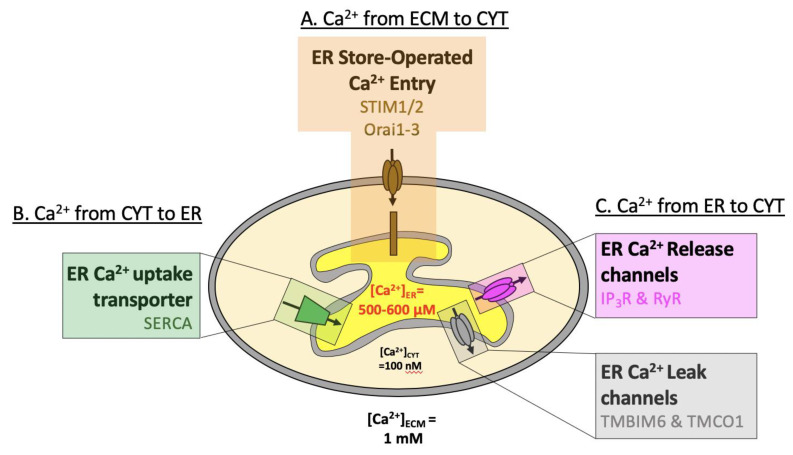
Overview of ER Ca^2+^ handling. Schematic presentation of Ca^2+^ concentrations in the endoplasmic reticulum (ER, 0.6 mM) compared to the cytosol (CYT, 100 nM) and extracellular milieu (ECM, 1 mM). (**A**) Ca^2+^ flows down its electrochemical gradient from the ECM to CYT (through Orai) or (**B**) against its electrochemical gradient from the CYT to the ER (through SERCA). (**C**) Ca^2+^ flows from the ER to the CYT down its electrochemical gradient, either following the activation of IP_3_R & RyR, or through ER Ca^2+^-leak channels TMBIM6 & TMCO1.

Morphological features distinguish the endoplasmic reticulum (ER) into the rough endoplasmic reticulum (RER) and the smooth endoplasmic reticulum (SER). Despite exhibiting morphological plasticity, RER presents itself in the form of flattened sheets whereas SER is mostly composed of tentacular tubules [11]. At the base of these morphological discrepancies are curvature-maintaining proteins. For instance, ER sheet formation involves the cytoskeleton-linking membrane protein 63 (CLIMP-63) and the transmembrane protein 170A (TMEM170A) while ER tubules are formed by reticulons (RTN1–4) and DP1/Yop1p family members [12]. Besides these morphological disparities, RER and SER execute distinct biological functions. The RER shares a common lumen with the nuclear membrane, which allows for the dynamic exchange of RNAs and proteins through nuclear pores [13]. Its cytoplasmic surface is “studded” with ribosomes where protein synthesis and modifications such as glycosylation occur [14]. Following entry into the RER through protein-conducting channels known as translocons, newly synthesized cytosolic proteins undergo extensive modifications such as folding, sorting, and even degradation in the case of protein misfolding [15]. Further away from the nucleus, the SER specializes in many metabolic processes, including lipid and steroid hormone synthesis, and cellular detoxification [2]. In addition, the SER operates as a major Ca^2+^ reservoir and is responsible for regulating ER Ca^2+^ dynamics which, in turn, ensure the optimal activity of both ER compartments. For instance, many chaperones exhibit high Ca^2+^ binding affinity and any perturbation in the concentration of ER Ca^2+^ impairs ER protein folding, leading to ER stress [16]. The SER Ca^2+^ dynamic affects the activation of both Ca^2+^ release (from the ER) and Ca^2+^ influx (from the extracellular space), generating cytosolic Ca^2+^ levels conducive to the activation of key Ca^2+^-dependent enzymes such as calcineurin, calmodulin-dependent kinases and/or binding proteins, all of which play important roles in proliferation, apoptosis, and motility [17]. Two defining features of the ER Ca^2+^ signaling network involve its ability to transmit localized, oscillatory Ca^2+^ signals throughout specific micro-domains within a cell and establish a compartmentalized Ca^2+^ gradient. Indeed, under resting conditions, the cytosolic Ca^2+^ concentration (≈100 nM) is 10,000 times lower than that of the extracellular space (≈1.5–2.5 mM) and 1000–5000-folds lower than that of the luminal ER (≈500 µM) (Figure 1) [18]. However, upon physiological stimulations, the cytosolic Ca^2+^ level rapidly increases from 100 nM to 1 mM either via Ca^2+^ release from its intracellular stores or via influx from the extracellular space [19]. Importantly, this rapid increase of cytosolic Ca^2+^ is followed by a timely Ca^2+^ removal system, re-setting the basis for Ca^2+^ homeostasis. The establishment of this Ca^2+^ gradient is vital in the functioning of all organisms and is coordinated by the activity of various ER Ca^2+^-releasing and-refiling transporters.

## 3. ER Ca^2+^-Releasing Channels

**Figure 2:**
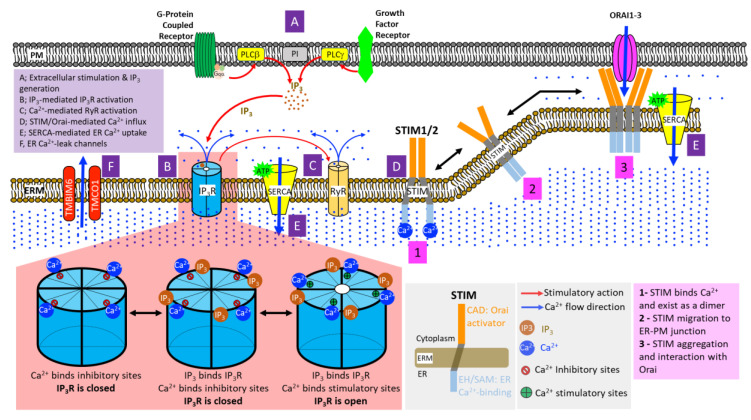
Mechanisms of ER Ca^2+^ handling. (**A**) Stimulations of G-protein Coupled Receptors (GPCRs) and Receptor Tyrosine Kinases (RTKs) signal to phospholipase C-beta (PLC-beta) and PLC-gamma at the plasma membrane, respectively. This leads to PLC-mediated hydrolytic cleavage of phosphatidylinositol 4,5-bisphosphate, producing the Ca^2+^-mobilizing inositol-1,4,5-trisphosphate (IP_3_) and diacylglycerol (DAG) (not shown in the figure) at the cell membrane. (**B**) Four molecules of IP_3_ bind to the tetrameric IP_3_ receptors (IP_3_Rs) on the ER membrane, exposing their stimulatory Ca^2+^ binding sites while simultaneously obstructing inhibitory Ca^2+^ binding sites. Upon the co-binding of Ca^2+^ and IP_3_, IP_3_R channel pore opens, initiating ER Ca^2+^ release. (**C**) Elevated cytosolic Ca^2+^ further induces the opening of ryanodine receptors (RyRs) on the ER membrane, causing rapid and massive influx of ER Ca^2+^ into the cytosol. (**D**) Dwindling luminal ER Ca^2+^ results in the oligomerization of EF-SAM domain of stromal interaction molecules (STIMs), which, in turn, induces the multimerization of cytoplasmic STIM domains followed by translocation and assembly of STIM clusters at the ER-plasma membrane (ER-PM) junctions. In direct physical association with Orai channels on the plasma membrane, STIM clusters induce the opening of Orai channel pore, allowing extracellular Ca^2+^ entry into the cytosol. (**E**) Powered by ATP hydrolysis, the Sarco/Endoplasmic Reticulum Ca^2+^-ATPases (SERCAs) shuttle the influx of extracellular Ca^2+^ into the ER or SR, restoring cellular Ca^2+^ homeostasis. (**F**) ER Ca^2+^-leak channels, such as TMBIM6 and TMCO1, prevent ER Ca^2+^ over-filling.

ER Ca^2+^ release into the cytosol begins with signals generated at the plasma membrane. Stimulation of G-protein coupled receptors (GPCRs) and receptor tyrosine kinases (RTKs) activates phospholipase C (PLC) beta and gamma, respectively. PLC then hydrolyzes the plasma membrane-enriched phosphatidylinositol 4,5-bisphosphate (PIP_2_) into 1,2-diacylglycerol (DAG) and inositol-1,4,5-trisphosphate (IP_3_) (Figure 2A). The newly generated IP_3_ diffuses into the cytoplasm and binds to its receptors (IP_3_ receptors, IP_3_Rs) on the ER membrane, causing them to open, ultimately leading to the release of ER Ca^2+^ into the cytosol (Figure 2B) [20]. The following sections will describe the functions and mechanisms of major ER Ca^2+^ transporters.

### 3.1. Inositol 1,4,5-Trisphosphate Receptors (IP_3_Rs)

IP_3_R is a macroscopic (~1.3 MDa), six-pass transmembrane ER Ca^2+^-release transporter, collectively functioning as a homo- or hetero-tetrameric assembly [21]. In humans, three genes sharing 70% sequence identity encode the three IP_3_R homologous isoforms, IP_3_R1s, IP_3_R2s, and IP_3_R3s [22]. IP_3_R1 is the most well-studied subtype and is ubiquitously expressed with the highest level detected in Purkinje neurons [23]. IP_3_R2 has the highest affinity for IP_3_ (IP_3_R2 > IP_3_R1 > IP_3_R3) and is predominantly expressed in hepatocytes [24,25,26]. Also broadly expressed, IP_3_R3 exhibits the highest expression level in gastric, salivary and pancreatic acinar cells [27]. Various mathematical and computational models have shed light on the gating kinetics of IP_3_Rs [28,29]. Despite those models being grounded on different assumptions, the prevailing view suggests that the tetrameric IP_3_R channels allow the binding of four IP_3_ molecules at the N-terminus and multiple Ca^2+^ ions at the C-terminus, differentially controlling the opening and closing of IP_3_Rs in a Ca^2+^ and IP_3_ concentration-dependent fashion [24,30,31]. Long-range allosteric regulation also exists to couple IP_3_-dependent conformational change at the N-terminus to the opening of Ca^2+^-conducting pore at the C-terminus [32]. Under resting conditions in the absence of bound IP_3_, IP_3_Rs direct the binding of Ca^2+^ to the inhibitory sites and remain closed. Upon stimulation, IP_3_ binds to IP_3_Rs and initiates channel opening by simultaneously exposing stimulatory Ca^2+^ binding sites and occluding the inhibitory Ca^2+^ binding sites, priming cytosolic Ca^2+^ to bind to the activating sites and facilitating ER Ca^2+^ release in a process known as Ca^2+^-induced Ca^2+^ release (CICR) (Figure 2B) [33]. Intriguingly, Yang and colleagues identified an IP_3_-independent caldendrin activation of all three IP_3_R channel isoforms [34]. Using gene editing to label endogenous IP_3_R1s and super-resolution microscopy, Thillaiappan et al. discovered that only a small population of immobilized IP_3_R clusters near the plasma membrane are licensed to respond to IP_3_ stimulation. These IP_3_R clusters initiate Ca^2+^ puffs that may serve as the origin and subsequent basis of both localized and distant propagation of CICR among the remainder dynamically-motile IP_3_Rs dispersed within the ER membrane [35,36]. Upon being expelled from the ER, IP_3_R-mediated Ca^2+^ transients are then selectively transported to various subcellular compartments including the cytosolic environment, the mitochondria through ER-mitochondria contact sites (ERMCS) (to which IP_3_R2 contributes the most), and the lysosomes via ER-lysosome contact sites [37,38,39]. Particularly, as cytosolic Ca^2+^ begins to rise, Ca^2+^ activates various downstream partners such as calmodulin, calcineurin and protein kinase C (PKC) which, in turn, modulate important cellular processes and functions, including transcriptional regulation, intracellular protein trafficking, differentiation, proliferation, adhesion and invasion. Ca^2+^ released to the vicinity of the plasma membrane can activate Ca^2+^-activated Cl^-^ channels, such as anoctamin 1 for heat sensing [40,41]. Furthermore, IP_3_R-mediated Ca^2+^ delivery to the mitochondria at the ERMCS serves as a pivotal signal for apoptotic induction and facilitates metabolic reprogramming [42,43]. Moreover, in addition to fine-tuning cytosolic Ca^2+^ oscillations, lysosomal sequestration of Ca^2+^ released by IP_3_Rs may affect the biological behavior of lysosomes, such as endo-lysosomal membrane trafficking [44,45]. In spite of the vastly heterogeneous IP_3_R isoform expression in most animal cells, all IP_3_R isoforms seem to generate, at least at the most rudimentary level among all Ca^2+^ signals, localized Ca^2+^ puffs with almost unifying puff amplitudes and spatial-temporal puff kinetics [46]. However, during prolonged stimulation and despite serving as the receptor’s co-agonist along with IP_3_, Ca^2+^ can trigger IP_3_R ubiquitination and subsequent degradation as a preventative measure against toxic buildup of cytosolic Ca^2+^ [47,48]. Introducing further complexity to the IP_3_R-mediated Ca^2+^ signaling landscape are the exquisite sensitivities to co-agonist Ca^2+^ across IP_3_R isoforms. For instance, IP_3_R1 and IP_3_R2 are under biphasic regulation by Ca^2+^ where a moderate increase in cytosolic Ca^2+^ enhances the response to IP_3_ stimulation while high cytosolic Ca^2+^ inhibits such response [49,50]. On the other hand, IP_3_R3 produces monophasic Ca^2+^ transients [51,52]. This distinct susceptibility of each IP_3_R isoform to modulation by varying levels of cytosolic Ca^2+^ may serve as an underpinning molecular mechanism for the regenerative nature of spatiotemporal Ca^2+^ signals that display diverse intensity, amplitude, and duration in normal physiology and disease states. Yet, the complexity of factors involved and the clinical significance of the channel crosstalk, cell-type isoform expression equilibrium, cellular distribution and receptor conformation of IP_3_Rs are still not fully understood.

### 3.2. Ryanodine Receptors (RyRs)

Located on the ER membrane, RyRs mediate massive and rapid Ca^2+^ release via CICR [53,54]. RyRs are normally closed at low cytosolic [Ca^2+^] ranging from 100–200 nM. Once the rising cytosolic [Ca^2+^] reaches a certain threshold, it begins to act on the RyRs, triggering the opening of the homo-tetrameric channels. Extensively characterized in excitable tissues, RyRs exhibit optimal opening probability at sub-micromolar cytosolic Ca^2+^ concentration [55]. As a result, the IP_3_Rs-mediated increase in the resting cytosolic Ca^2+^ concentration paves the way for RyRs to reach their maximum functional capacity. Due to the critical nature of high-conductance RyRs in maintaining cellular electrophysiology, many proteins and molecules, such as calmodulin, calmodulin-dependent protein kinase II (CaMKII), Protein Kinase A (PKA), nicotinamide adenine dinucleotide hydrogen (NADH) and Mg^2+^, contribute to the precise functional modulation of this supramolecular assembly [56,57,58,59].

The roles of IP_3_Rs and RyRs are not confined to merely facilitating Ca^2+^ efflux. Seemingly trivial, the diverse forms of Ca^2+^ signaling are encoded in spikes, sparks, blips, puffs, and waves, often having profound biological implications. For instance, Ca^2+^ spikes at the micro-molar range are commonly observed in the apical pole of pancreatic acinar cells to assist in limited exocytosis and secretion of zymogen granules in response to low IP_3_ stimulation [60,61]. In cardiac myocytes, the opening of voltage-gated L-type Ca^2+^ channels caused by membrane depolarization increases intracellular Ca^2+^ level, triggering the opening of RyR2 and subsequent Ca^2+^ sparks, an essential element in maintaining excitation-contraction coupling in healthy cardiac functions [62]. Moreover, Ca^2+^ blips, also known as “triggering events” for Ca^2+^ puffs, are formed by small, transient Ca^2+^ elevations associated with the opening of a single IP_3_R channel. A multitude of single IP_3_R channels in an IP_3_R cluster evokes localized elevation of Ca^2+^ resulting in puffs which can affect nuclear Ca^2+^ signaling through fine-tuning Ca^2+^ delivery into nucleoplasm and potentially transcription [63]. With higher stimulation from IP_3_, Ca^2+^-induced Ca^2+^ release becomes an activating ligand for one cluster site to drive Ca^2+^ release at adjacent sites, leading to the generation of Ca^2+^ waves that propagate in a saltatory manner [64]. The generation of spatially confined Ca^2+^ waves has been linked to the modulation of the disassembly and turnover of focal adhesion sites, a process highly exploited during cancer metastasis [65]. A linear correlation between IP_3_R cluster size and Ca^2+^ puffs has also been established, suggesting that large clusters are potentially responsible for carrying out pacemaker activities [66]. Adding complexity to the already intricate network of Ca^2+^ signaling is the incorporation of yet another positive feedback mechanism that couples oscillations of Ca^2+^ to oscillations of IP_3_, all mediated by phospholipase C [28]. Together, IP_3_Rs fine-tune ER Ca^2+^ release whereas RyRs amplify such a response, effectively elevating cytosolic Ca^2+^ at a global scale.

## 4. ER Ca^2+^ Replenishment

As IP_3_Rs and RyRs act synergistically to increase the cytosolic Ca^2+^ concentration in order to mediate downstream signal transduction, the ER responds to its dwindling Ca^2+^ repository by activating store-operated Ca^2+^ entry (SOCE). The following paragraphs will detail the major players responsible for the ER cytoplasmic Ca^2+^ refill.

### 4.1. STIM-Orai

To mediate store-operated Ca^2+^ entry, the stromal interaction molecules (STIMs) located on the ER membrane physically interact with and activate Ca^2+^-selective Orai channels at the plasma membrane to mediate Ca^2+^ influx from the extracellular space. This phenomenon is known as Ca^2+^ release-activated channel (CRAC) [67,68]. STIM, a single-pass transmembrane protein, senses ER Ca^2+^ level using its luminal EF-hand and sterile alpha-motif (EF-SAM) domain and functions as the primary initiator of SOCE [69]. In the presence of ample ER Ca^2+^, the luminal EF-SAM of STIM1 is loaded with Ca^2+^ and exists in a monomeric state. Upon ER Ca^2+^ depletion, however, the luminal EF-SAM domain undergoes a conformational change that allows it to become aggregated and capable of directing the cytoplasmic portion of STIM to oligomerize and assemble at the ER-plasma membrane (ER-PM) junctions to be in close physical proximity with Orai channels [69]. Signaling through its STIM-Orai activating region (SOAR), STIM1 induces Orai channel opening and triggers Ca^2+^ influx from the extracellular space [70]. Considering the critical role of CRAC channel activity in maintaining the healthy dynamics of ER Ca^2+^ signaling, multiple safety mechanisms are established to ensure the functional regulation of CRACs (Figure 2E). For instance, STIM-induced ER-PM junctional domains contain regulatory proteins, such as CRAC regulatory protein 2A (CRACR2A), junctate, and partner of STIM1 (POST), to fine-tune Ca^2+^ mobilization into the cytosol [71]. To prevent excessive Ca^2+^ entry, STIM2.1, a naturally occurring STIM2 variant, hinders STIM-Orai cross-linking and decreases clustering of CRAC channels at the plasma membrane [72]. Furthermore, shifting away from over-reliance on the STIM-Orai mediated Ca^2+^ entry, transient receptor potential vanilloid 6 (TRPV6) has been reported to translocate from the ER to the plasma membrane to supply Orai-mediated Ca^2+^ influx [73]. Together, the existence of intricate regulatory networks for CRACs and the functional multiplicity underlying Ca^2+^ entry following ER Ca^2+^ depletion equip cells to battle Ca^2+^ fluctuations in times of stress.

### 4.2. SERCAs and ER Ca^2+^-Refilling

As STIM-Orai initiates Ca^2+^ influx from the extracellular space, the Sarco/Endoplasmic Reticulum Ca^2+^-ATPase (SERCA) provides a means for excess cytoplasmic Ca^2+^ to be shuttled and stored into the ER, establishing a 1000-fold [Ca^2+^] gradient between the ER and cytosolic compartments [74]. A member of the P-type ATPase superfamily, SERCA utilizes the energy from ATP hydrolysis to alternate between two conformations, E1 and E2 each binding two Ca^2+^ with high specificity from the cytoplasmic side and releasing them into the luminal ER and SR, respectively [75]. In humans, the SERCA pump is encoded by three genes, *ATP2A1*, *2* and *3*. Post-transcriptional modifications, mainly alternative splicing, generate at least 14 SERCA variants with diverse species-dependent cellular and tissue distributions throughout various stages of development [76,77,78,79]. The level of SERCA1a is the highest in adult slow-twitch skeletal muscles whereas SERCA1b is found predominantly in fetal fast-twitch muscles. In contrast, *SERCA2a* is expressed in cardiac tissues while *SERCA2b* is ubiquitously expressed. SERCA3 variants are often found co-expressed with the SERCA2b variant in a wide variety of tissues and cells, such as the salivary glands, lymphoid tissues, pancreatic cells and cerebellar Purkinje neurons [80,81]. The housekeeping SERCA2b protein, for instance, is composed of 3 cytosolic (A, N, and P) domains responsible for mediating ATP binding and hydrolysis, and one 11-helix transmembrane region involved in the regulation of Ca^2+^ transport [82,83]. As there seems to be notable differences in Ca^2+^-binding affinities across SERCA isoforms and amongst variants within the same SERCA isoform, the tissue-specific expression equilibrium of SERCA variants transmits differential Ca^2+^ rhythms required for the survival and function of that specific tissue [84,85]. Considering the crucial role of SERCA pumps in maintaining ER Ca^2+^ homeostasis, the intricate modulatory mechanisms and existence of various SERCA variants allow for a tight control of the molecular dynamics and kinetic behavior of this pump [86]. Furthermore, SERCA activity is modulated by various factors. For instance, curcumin presumably inhibits SERCA by preventing ATP binding leading to the inhibition of ATP-dependent ER Ca^2+^ uptake, whereas phospholamban (PLB) and its homolog sarcolipin act by reducing SERCA’s affinity for Ca^2+^ through direct interaction with SERCA at several ER transmembrane sites [87,88,89,90].

To prevent ER Ca^2+^ over-filling and ER stress, the transmembrane BAX inhibitor motif containing protein (TMBIM) and the transmembrane and coiled-coil domain 1 (TMCO1) act as Ca^2+^-leak channels. Among the six TMBIM protein family members, strong evidence points to TMBIM6 (or BI-1) being a seven-pass, pH-sensitive Ca^2+^-leak channel strictly localized to the ER in skeletal muscle, liver, kidney and spleen [91,92]. Structural insights on the bacterial homolog BsYetJ revealed that the distinct protonation states of Asp171 under acidic and alkaline pH environments affecting hydrogen bonding dynamics among Arg60 on the transmembrane 2 (TM2) region and the C-terminal di-aspartyl pH sensor Asp171 and Asp195 are responsible for altering the positioning of TM2 to mediate Ca^2+^ fluxes across membranes [93,94,95]. This is in agreement with previous finding stating the indispensable role of Asp213 (human equivalent of Asp195 in BsYetJ) in authorizing Ca^2+^ fluxes of synthetic human C-terminal peptide of BI-1 [96]. In addition to the passive Ca^2+^-leak channel TMBIM6, TMCO1 is a Ca^2+^ load-activated Ca^2+^ (CLAC) channel embedded across the ER membrane [97]. Mechanistically, TMCO1 undergoes reversible homo-tetramerization upon ethanol-induced elevation of ER Ca^2+^ content, forming a Ca^2+^-selective channel to allow the extrusion of excess Ca^2+^ before it rapidly dissembles upon restoration of the resting luminal ER [Ca^2+^]. The dynamic orchestration of Ca^2+^ uptake and release contributes to the maintenance of ER Ca^2+^ homeostasis, protecting the ionic integrity of the ER for cellular survival and physiological functions.

## 5. ER Ca^2+^ Transporters and Cancer Pathophysiology

The pitfalls of abnormal levels of activity of the ER Ca^2+^ transporters are manifested clinically in a diverse array of human cancers. As tumor pathogenesis varies with each malignancy, it is important to be aware of the highly context-dependent nature of the methods through which cancer cells hijack the ER Ca^2+^ signaling. The rest of this review will cover some of the mechanisms employed by cancer cells to sabotage ER Ca^2+^ signaling and the current therapeutic strategies being investigated as potential treatments for cancer patients.

### 5.1. IP_3_Rs in Cancer

The strategic positioning and close proximity of the ER to key organelles (e.g., mitochondria, lysosomes and nucleus) have allowed IP_3_Rs to emerge as crucial determinants of cell fate [98,99]. As a result, IP_3_Rs must strike a meticulous balance among allocating and transferring appropriate levels of Ca^2+^ into the mitochondria to ramp up cellular bioenergetic supplies, into the lysosomes to modulate autophagy, and into the nucleus to regulate transcription. Complex regulations of IP_3_Rs have been documented, preponderant insights of which come from the pro-apoptotic and anti-apoptotic members of the B-cell lymphoma 2 (Bcl-2) family proteins that act primarily by affecting mitochondrial membrane permeability. Within the human Bcl-2 family, pro-apoptotic members (Bax, Bak, Bok, Bid, BAD, Bik, Bim, Noxa, PUMA) can be distinguished by the acquisition of the Bcl-2 homology 3 (BH3) domain, whereas the anti-apoptotic proteins (Bcl-2, Bcl-XL, Mcl-1, Bcl-W, BFL-1, Bcl-B) not only include this BH3 domain but also harbor the Bcl-2 homology 4 (BH4) domain at the N terminus to keep cellular apoptosis at bay [100,101]. Some of the prominent ways in which anti-apoptotic Bcl-2 proteins hijack ER Ca^2+^ signaling to minimize the production of apoptotic Ca^2+^ transients are based on protein-protein interactions. Using Fluorescence Resonance Energy Transfer (FRET) and GST-IP_3_R1 fragment pulldowns, Rong and colleagues precisely pinpointed that endogenous Bcl-2 binds to amino acid residues 1389–1408 in the regulatory and coupling domain of IP_3_R1 to inhibit its apoptosis-inducing Ca^2+^ release in Jurkat cells [102]. In a subsequent study, Rong et al. defined the BH4 domain of the Bcl-2 protein to be a functional unit that conferred anti-apoptotic protection against IP_3_R1 activity [103]. Furthermore, using bioinformatics and site-directed mutagenesis, Monaco and colleagues discovered that a single amino acid difference in the BH4 domain of Bcl-2 and Bcl-XL may account for the differential binding of the proteins to IP_3_R1 and the distinctive regulation of IP_3_-induced Ca^2+^ release (IICR) [104]. Recent evidence suggests that, in addition to binding to the modulatory region of the IP_3_R1 as Rong et al. proposed in 2008, purified BH4 domain of Bcl-2 is also capable of forming a physical complex with and participating in competitive binding to the ligand-binding domain (LBD) of IP_3_R1 with receptor agonist IP_3_ to either activate or inhibit the IP_3_R1 channel activity in concordance with the extent of IP_3_-evoked receptor stimulation [105]. As Bcl-2-IP_3_R interaction was established, scientists began to search for the potential involvements and mechanistic understandings of other anti-apoptotic Bcl-2 family members in the modulation of IP_3_R Ca^2+^ release. Interestingly, in stark contrast to Bcl-2 inhibition of IP_3_R channel activity, Bcl-XL sensitizes all three IP_3_R isoforms to IP_3_ stimulation while promoting ER- Ca^2+^-mediated mitochondrial bioenergetics and enhancing spontaneous cytosolic Ca^2+^ signaling in conferring apoptotic resistance [106,107]. However, new structural evidence uncovers that the biphasic regulation of IP_3_R channel gating kinetics in the maintenance of cell viability occurs through the binding of BH3-like domain on the carboxyl terminus of IP_3_R by the BH3 domain-binding pocket of Bcl-XL [106]. Indeed, structurally similar anti-apoptotic Bcl-2 proteins, such as Mcl-1, have been reported to bind to the carboxyl termini of all three mammalian IP_3_R isoforms with comparable affinity and increase spontaneous IP_3_R-dependent Ca^2+^ oscillations as necessary steps to maintaining cellular survival in response to cytotoxic agents [108]. Nonetheless, these complexes of anti-apoptotic Bcl-2 proteins and IP_3_R channels may open the door for innovative therapeutic interventions. As a matter of fact, recent years have witnessed the tremendous breakthrough in the use of synthetic peptides to disrupt the Bcl-2-IP_3_R complex in chronic lymphocytic leukemia, multiple myeloma, follicular lymphoma and small cell lung cancer either alone or with other mimetics to potentiate anti-neoplastic effects and / or tackle chemo-resistance [109,110,111,112,113]. On the other end of the spectrum, a growing body of evidence suggests that IP_3_R activity is subject to regulation by tumor suppressors. For instance, tumor suppressor proteins phosphatase and tensin homolog (PTEN) in human prostate cancer and BRCA1-associated protein-1 (BAP1) in asbestos-induced malignant transformation partially act through stabilizing IP_3_R3s against receptor ubiquitination; thus, potentiating Ca^2+^ transport into the mitochondria to drive apoptosis [114,115]. Furthermore, in colorectal cancer cell lines, abrogation of oncogenic K-Ras unleashed IP_3_R3 activity, enhancing IP_3_R3-mediated Ca^2+^ release and inducing cellular sensitization to apoptosis [116]. As a result, the systematic coordination of these effector regulators of IP_3_Rs carries profound impacts on cell fate.

Besides the functional regulation of IP_3_Rs by the oncoprotein-tumor suppressor crosstalk, the selective expression of individual IP_3_R isoform has also been tampered with in several clinical malignancies. For instance, IP_3_R3 is up-regulated in gastric cancer, glioblastoma and renal cell carcinoma [117,118,119]. Additionally, *IP_3_R3* expression level has been found increased and positively correlated with the migratory and invasive capacities of breast cancer and glioblastoma cell lines and that caffeine-mediated IP_3_R3 inhibition abrogated proliferative and invasive phenotypes in glioblastoma and extended survival rate [120,121]. As migration is associated with cell shape, IP_3_R3 likely remodels cytoskeletal structure to support breast cancer cell migration and invasion [122]. Moreover, surgically resected colorectal carcinomas indicated elevated *IP_3_R3* expression, in proportion to the depth of invasion, lymph node and liver metastases [123]. Collectively, these evidence makes elevated IP_3_R3 level a reliable diagnostic marker for various clinical malignancies. Unlike IP_3_R3, whose expression pattern has been well documented in human carcinomas across multiple tissues, the expression profiles of IP_3_R1 and IP_3_R2 remain elusive in pathophysiology as they seem to draw diverse implications on various aspects of tumorigenesis, such as tumor initiation, migration, survival, and even drug resistance. For instance, heightened IP_3_R1 activity promotes prostate cancer cell survival and resistance to hormonal deprivation therapy [123]. Conversely, increased IP_3_R1 level is shown to potentiate melatonin-induced apoptosis among ovarian cancer and colorectal cancer cell lines while simultaneously conferring attenuated antioxidant responses [124]. Similarly, this pro-apoptotic effect of IP_3_R1 has also been studied in vitro and in vivo after subjecting ovarian carcinoma cells to cytotoxic agent sulforaphane [125]. Moreover, *IP_3_R1* expression is markedly reduced in cisplatin-resistant bladder cancer cell lines and that transient induced over-expression of *IP_3_R1* in resistant cells restored chemo-sensitivity to cisplatin [126]. Exerting similarly broad impacts as IP_3_R1s, IP_3_R2-mediated Ca^2+^ oscillation plays extensive roles ranging from lung cancer cell migration, maintenance of the regenerative capacity of liver cancer stem cells and to the induction of senescence [127,128,129]. Moreover, recent study shows that, diffuse large B-cell lymphoma (DLBCL) cells with constitutive IP_3_ signaling and addiction to Bcl-2-mediated attenuation of IP_3_R2 Ca^2+^ release are sensitive to apoptotic induction by Bcl-2/IP_3_R Disruptor-2 (BIRD2), which is compatible with the previous finding in DLBCL that increased IP_3_R2 protein level is associated with high sensitivity to apoptosis among SU-DHL-4 cells subsequent to treatment with BIRD2 [130,131]. Collectively, rampant manipulations of the IP_3_R expression profile throughout cancer development epitomize the notion that many malignancies have harbored the increasingly diversifying capacity to sabotage IP_3_R-mediated Ca^2+^ transients and therefore, global Ca^2+^ signaling to stimulate oncogenesis at the genetic level.

### 5.2. RyRs in Cancer

Encoded by three separate human genes and composed of homo-tetrameric supramolecular assemblies, RyRs (I, II, and III) are mostly expressed and studied in the context of excitable tissues, including skeletal muscle, cardiac tissues, and the brain, respectively [132,133,134]. However, emerging clinical and empirical evidence from oncological studies has described the functional expression of RyRs as highly diverse across a vast array of human malignancies. For instance, Abdul et al. examined the total RyR protein expression in patient-derived ductal breast cancer epithelium and found that the overall RyR expression is positively correlated with tumor grade, suggesting the involvement of RyRs in breast cancer survival. However, the addition of RyR agonist, 4-chloro-m-cresol, inhibited breast cancer cell proliferation [135]. Furthermore, in comparison with normal thyroid tissues, tissues derived from thyroid carcinoma exhibit decreased expression of *RyR2*, the down-regulation of which is tightly associated with decreased patient survival rate, lymphatic metastasis, extracapsular extension, and bleak clinical prognosis [136]. On the other hand, *RyR2* is over-expressed in melanoma tissues as compared to melanocytes. However, the reported increase of *RyR2* expression is not concomitant with an increase in RyR-mediated Ca^2+^-release [137]. Similar results were reported by Bennett and colleagues, who demonstrated that neither ryanodine nor caffeine (RyR agonist) elicited a measurable RyR2-mediated Ca^2+^ transient in cervical cancer epithelial cell line HeLa, suggesting aberrant functional properties of RyR2s in the survival of cancer cells [138]. Besides aberrant *RyR2* expression levels in giving rise to malignancy, several mutations of RyR2s have been linked to lung cancer [139]. Furthermore, *RyR3* over-expression is detected in breast cancer where RyR3s play an essential role in proliferation and migration [140]. Nevertheless, studying RyRs through the lens of cellular apoptosis, Mariot et al. demonstrated that the functional expression and activation of RyR1s and RyR2s by caffeine led to apoptosis of prostate cancer LNCaP cells, whereas inhibition of these receptors with ryanodine protected against apoptosis [140]. Furthermore, even with apoptosis-resistant cancer cells, RyR-mediated Ca^2+^ release has been shown to facilitate Neferine-induced autophagic cell death [141].

In addition to mediating cancer progression, RyRs have also been linked to chemo-resistance. For instance, RyR1s contribute to acquired chemo-resistance by executing non-enzymatic interactions with chemotherapy-induced GSTO1 (glutathione S-transferase omega 1) to fine-tune cytosolic Ca^2+^ levels needed for the enrichment of the tumor-initiating breast cancer stem cells (BCSCs) [142]. Though recognizing RyR1′s role in driving BCSCs seems promising in tackling chemo-resistance, the feasibility of achieving pharmacological inhibition of RyR1s remains low due to limitations imposed by drug delivery, resultant toxicity, and target specificity across RyR subtypes [143]. While current findings hold promise for the derivation of a future RyR-based anti-neoplastic therapy, more research is needed to understand the underlying mechanisms of the differential regulation of these receptors in physiology and pathophysiology.

### 5.3. STIM-Orai Channels in Cancer

Ca^2+^ signaling sets the fundamental basis for metastatic dissemination of tumors to distant tissues through activation of proliferative and invasive pathways, such as nuclear factor of activated T-cells (NFAT) and extracellular signal-regulated kinases (ERKs) [144,145]. The activity of these oncogenic pathways is often dictated by pathological modifications of Ca^2+^ release and influx channels, in particular, at the level of store operated Ca^2+^ entry (SOCE). Since STIM1-Orai1 signaling axis encompasses the predominant mechanism underlying SOCE, it is often the target of oncogenic manipulations at both genetic and functional levels. For example, *STIM1* is over-expressed in colorectal cancer and its expression level is positively correlated with tumor size, depth of invasion, and lymph node metastasis [146]. In glioblastoma multiforme, *STIM1* and *Orai1* knockdown decreased cancer cell invasion and proliferation, respectively [147]. STIM1 is also a crucial mediator for cell proliferation, migration as well as angiogenesis in cervical cancer and invasion in melanoma [148,149]. Furthermore, in breast cancer cell line MDA-MB-231, STIM1 and Orai1 remodel focal adhesion turnovers and are required for tumor invasion and metastasis [147]. Similar observations were ascertained in pancreatic ductal adenocarcinoma where STIM1-mediated ER-PM junction formation was found to be re-distributed during epithelial-mesenchymal transition, underscoring the essential role of altered state of SOCE in cellular migration and malignant transformation [150]. Furthermore, revealed by time-lapse imaging, esophageal squamous cell carcinoma (ESCC) KYSE-150 cells showed hyperactive spontaneous intracellular Ca^2+^ oscillations, potentially due to the elevated expression of *Orai1* [151]. To corroborate this, McAndrew further demonstrated that *Orai1 siRNA* knockdown not only attenuated cytosolic Ca^2+^ influx in breast cancer MDA-MB-231 and MCF-7 cell lines in the presence of invasive stimulus PAR-2, but also reduced their viability [152]. As *STIM1* and *Orai1* over-expression has been observed across a multitude of malignancies, they are inarguably among the most enticing drug targets in anti-cancer therapy.

In addition to adjusting the expression and activity of the canonical STIM1-Orai1 signaling axis, cancer cells also have developed the ability to switch to store-independent Ca^2+^ entry to escape a potentially “doomed fate.” It was not until the 1990s that an alternative “store-independent Ca^2+^ entry” model was proposed to provide a more accurate depiction of Ca^2+^ entry under a physiological level of agonists. The proposed mechanism suggested that, instead of sustained elevated intracellular Ca^2+^, subtle periodic oscillations of intracellular Ca^2+^ take over during SOCE [153,154]. Although Orai1 was the most well understood ion channel at the time, the possibility of alternative mechanisms responsible for such periodic oscillations of Ca^2+^ entry led scientists to examine the functions and roles of other members of the Orai family channels. Motiani et al. explored the selective requirement of many breast cancer cell lines for the use of Orai3 as opposed to the canonical Orai1-mediated SOCE based on the presence or absence of plasma membrane estrogen receptors [155]. Later, in 2013, Motiani et al. further demonstrated the selective use of Orai3 Ca^2+^ channels in mediating SOCE in estrogen receptor α-expressing (ERα^+^) breast cancer cells. Conversely, Orai3 knockdown led to decreased ERα^+^ MCF7 cell proliferation and invasion [156]. Another independent study led by Faouzi also demonstrated that *Orai3* knockdown impaired breast cancer MCF-7 cell proliferation and arrested cell cycle progression at the G1 phase without affecting the proliferation and survival of wild-type mammary MCF-10A cells [157]. As the role of Orai3 in facilitating tumorigenicity became more prominent, Dubois et al. discovered increased endogenous expression of Orai3 protein and increased reliance on the use of Orai3-Orai1-jointly-mediated store-independent, arachidonic-acid-regulated channels among prostate cancer cells. This selective utilization of Orai3 by prostate cancer cells can partially be attributed to greater evasion of apoptotic signals closely associated with sole Orai1 functioning [158]. This change in the Orai3/ Orai1 expression dynamic created a shift from the use of the canonical Orai1-based SOCE and marks the oncogenic switch that facilitates prostate cancer tumor progression. The remarkable capacity of cancer cells to adjust their receptor expression equilibrium to enhance survival while achieving the same biological signaling outputs is a truly fascinating area for scientific investigation and a promising realm for drug discovery.

### 5.4. SERCAs in Cancer

SERCA activity represents a nodal point of cellular survival and has been extensively exploited in carcinogenesis [159]. The expression profile of SERCA I, II, and III isoforms is highly diverse across human malignancies. Mounting evidence indicates that many SERCAs are down-regulated in cancer. For instance, the SERCA1 isoform is decreased in cisplatin-resistant epithelial ovarian cancer cell line MDAH-2774 [160]. *SERCA2b* expression is significantly reduced in small cell lung cancer, thyroid cancer, oral squamous cell carcinoma and colon cancer [161,162,163,164]. Additionally, highlighting the interplay between SERCA2 deficiency to malignancy came the finding of Prasad et al. that haploinsufficiency of *Atp2a2*, which encodes the SERCA2 isoform, leads to increased likelihood of developing squamous cell papillomas [165]. Furthermore, the level of SERCA3 isoform plummets in breast carcinomas and colon adenocarcinomas [166,167]. An in-depth mechanistic explanation as to why SERCA down-regulation takes prevalence in these types of cancer is, nevertheless, still lacking. Considering SERCAs function by selectively replenishing the ER Ca^2+^ store, a pivotal biological implication connecting decreased luminal ER Ca^2+^ re-filling and cancer cell apoptotic resistance suggests that reduced ER Ca^2+^ store, despite exerting pleiotropic effects on intracellular Ca^2+^ handling, may translate into low cytosolic Ca^2+^ release, therefore, decreased activity of Ca^2+^-induced opening of the mitochondrial permeability transition pore (PTP), hence greater cell survival [168,169,170,171]. Supporting this notion, many research endeavors have found that the anti-apoptotic Bcl-2 protein upregulated in numerous malignancies inhibits the activity of various SERCA isoforms, leading to reduced ER Ca^2+^ uptake and attenuated pro-apoptotic mitochondrial Ca^2+^ influx [172,173]. Similarly, Scorrano et al. demonstrated that double knockout of pro-apoptotic Bcl-2 family members, *Bax* and *Bak* in mouse embryonic fibroblasts resulted in the inhibition of SERCA activity and decreased mitochondrial Ca^2+^ uptake, depicting a delicate balance between anti-apoptotic and pro-apoptotic Bcl-2 family members in fine tuning ER Ca^2+^ release [174]. Nonetheless, the complete and irreversible abolishment of SERCA activity by thapsigargin (TG) drives intrinsic apoptosis through the induction of prolonged ER stress [175,176]. Of note, many chemotherapeutics act through tumor suppressors to modulate Ca^2+^ signaling. A prominent example of this is that in response to Adriamycin challenge, the master tumor suppressor protein p53 localizes to the ER/mitochondria associated membranes and promotes SERCA activity by reducing its oxidation. This gives rise to ER Ca^2+^ overload and elicits ER Ca^2+^ release as a means of apoptotic induction [177]. Importantly, revealed by intravital fluorescent microscopy, this critical crosstalk between the SERCA pump and p53 in generating apoptotic signals is also substantiated in vivo in cancer photodynamic therapy using light-activated photosensitizer phthalocyanine, linking p53 sensitization of cellular apoptosis to ER Ca^2+^-overload and increased mitochondrial Ca^2+^ uptake [178]. Intriguingly, an alternative paradigm argues that SERCA over-expression has also become a hallmark in a variety of cancers. For instance, *SERCA2b* expression is positively correlated with colorectal malignancy as *SERCA2b* over-expression promotes pro-survival mitogen-activated protein kinase (MAPK) and protein kinase B (also known as AKT) signaling and drives proliferation and migration of human colorectal adenocarcinoma SW480 cells [179]. Moreover, *SERCA2b* is found over-expressed in epithelial prostate cancer cells and that knockdown of *SERCA2b* decreases prostate cancer proliferation [180]. In comparison with normal cells treated with curcumin, curcumin inhibition of SERCA2 activity selectively inhibits ovarian cancer cell viability [181]. Furthermore, upregulation of the SERCA3 isoform is detected in gastric carcinomas [182]. Mechanistically, by increasing luminal ER [Ca^2+^] via *SERCA* over-expression, rapidly proliferating cancer cells strategically endure and alleviate cytotoxic stress associated with their hyperactive protein synthesis and folding machineries [183]. Since cancer is a multifactorial disease, it is not surprising that even a combination of SERCA2(b) up-regulation and SERCA3 isoform down-regulation exists in the case of epidermal growth factor-induced epithelial mesenchymal transition in breast cancer MDA-MB-468 cells, further solidifying the link between aberrant SERCA activity and malignancy [184]. The purpose of reprogramming the expression pattern of SERCA isoforms in various malignant lesions is to confer cancer cells the ability to tailor the amplitude, duration and frequency of ER Ca^2+^ re-uptake to sustain their specific oncogenic needs. Hence, it is within reason that different SERCA isoforms demonstrate varying expression kinetics throughout distinct stages of tumorigenicity [185].

## 6. Targeting ER Ca^2+^ Signaling in Anti-Cancer Therapy

As ER Ca^2+^ signaling is indispensable for cell development, movement, metabolism, survival, and signal transduction, this, therefore, poses a challenge for a specific and efficacious Ca^2+^-based drug design. Similar to the “undruggable” Ras and MAPK, targeting ER Ca^2+^ alone seems impractical due to its ubiquitous presence and integral contribution to cellular physiology [186]. However, targeting proteins that interact with Ca^2+^ at the levels of channels/transporters/pumps and downstream effector molecules that decipher Ca^2+^-encoded messages seems to be more feasible [187]. Rather than targeting the IP_3_R channel activity alone as an isolated molecular entity, research is now directed towards gaining collective understandings of the fate-determining pathways following IP_3_R -mediated Ca^2+^ release, such as the IP_3_R-VDAC1-MCU-signaling axis bridging ER Ca^2+^ release and mitochondrial Ca^2+^ uptake [188]. Indeed, many chemotherapeutic agents, such as cisplatin and doxorubicin, fine tune ER-mitochondria crosstalk and alter oncogene-tumor suppressor function dynamics to elicit potent apoptotic Ca^2+^ signals, inhibiting tumor cell survival [189]. It is not the intention of this review to cover all channel inhibitors governing ER Ca^2+^ signaling, however, we will briefly describe the use of ER Ca^2+^ transporter-based chemical drug conjugate, computational pharmacology and extrapolate the immunotherapeutic potential of CRAC channels in the design of novel anti-neoplastic therapy.

Conjugating tumor-specific marker with seemingly unlikely drug target offers new hope in drug delivery. For instance, thapsigargin (TG) is widely used in research laboratories to deplete ER Ca^2+^ through prolonged inhibition of SERCA activity. This depletion of the ER Ca^2+^ store, in and of itself, induces ER stress, and causes elevated cytoplasmic Ca^2+^ that can activate intrinsic apoptotic pathways through calmodulin/calcineurin-mediated signal transduction [190]. Despite being shown to potentiate taxane-mediated tumor killing, TG has not been widely adopted in clinical settings due to its non-selective cytotoxicity [191]. Recognizing the heterogeneous molecular signatures of a tumor would vastly boost our chance of designing targeted therapies. An example of such attempt was documented by Denmeade and Isaacs: “Chemical modification and coupling of thapsigargin to a PSA-cleavable peptide sequence carrier seems to be a promising approach to target both normal and malignant prostate cancers” [192]. This pro-drug construct allows for the specific delivery of TG to prostate cancer cells, disrupting ER Ca^2+^ signaling and generating ER stress to induce apoptosis.

As an alternative investigative tool, computational pharmacology has been utilized to explore Ca^2+^ binding kinetics during SOCE. Found up-regulated in glioblastoma multiforme (GBM), STIM1 and Orai1 are positively associated with GBM invasiveness [193]. Through the use of in-silico models, such as molecular dynamic simulations and structure-based virtual screening, Sampath and Sankaranarayanan identified SB01990, SPB06836, and KM06293 as drug leads capable of disrupting Ca^2+^ binding to the active sites of Orai1, inhibiting ORAI-mediated Ca^2+^ influx with relatively ideal pharmacokinetics [194]. However, further in vivo testing is required to characterize the pharmacodynamic and pharmacokinetic properties of those inhibitors.

Another interesting area for clinical implementation of CRAC-channel based drug design is immunotherapy. Within a tumor mass, the tumor microenvironment (TME) is produced by the functional crosstalk among miscellaneous cell types, such as the cytotoxic T-lymphocytes (CTLs), B-lymphocytes, Natural Killer (NK) cells, tumor-associated macrophages (TAMs), tumor-associated neutrophils, regulatory T cells, pericytes, vascular endothelial cells, and cancer-associated fibroblasts [195,196,197]. Among these cells, CTLs and NK cells primarily exert their anti-tumor effect by secreting granzymes and perforin directly into the tumor cells resulting in cell lysis. Considering the critical involvement of STIM-Orai channels in the production of Ca^2+^ transients required for the proliferation, migration, recruitment of T lymphocytes as well as the subsequent degranulation of lytic vesicles into the tumor cell, CRAC channel activity is key to the initiation and maintenance of a potent anti-tumor immune response [198,199,200]. Indeed, loss-of-function mutations in human ORAI1 or STIM1 lead to increased susceptibility of developing tumors [201]. However, considering that CRAC channel activity is essential for both anti-tumor immunity and oncogenesis (as discussed in Section 5.3), it is, therefore, essential to acknowledge the double-edged-sword effect of utilizing STIM and Orai proteins as a therapeutic axis and that a comprehensive understanding of tumor-specific channel regulators and downstream signaling pathways is needed before therapeutic design.

Adding to the complexity of targeting ER Ca^2+^ signaling for cancer therapy are the different facets of drug design. What are the precise pharmacophore and mechanisms of action? Are there any non-specific interactions with other drug molecules, targets, or enzymes? What are the pharmacokinetic properties, namely absorption, distribution, metabolism, and excretion associated with the drug? Are there adverse side effects? How to reconcile and fit thermodynamic stability, drug bioavailability, and solubility all into the diverse pharmacogenomics of the patients? Nonetheless, we are entering an exciting era of biomedical research where basic mechanistic understandings of ER Ca^2+^ and its homeostatic regulation are vigorously pursued for the development of new anti-cancer therapies.

## 7. Closing Remarks

In this review, we have summarized the major ER Ca^2+^ transporters and their aberrant functional alterations in cancer. We described the homeostatic regulation of ER Ca^2+^ store and its connection with the global Ca^2+^ signaling transduction network. We also appreciated the many ways ER Ca^2+^ signaling manifests itself through its receptor distribution, isoform expression, downstream effector landscape and how these processes could be hijacked in malignancies. We examined the dynamic modulation of these transporters in the context of organellar crosstalk as well as endogenous regulation by oncoproteins and tumor suppressors. We then culminated the review with pharmacological interventions of ER Ca^2+^ transporters. As future design of anti-cancer therapy continues, it is awe-inspiring to reflect on the width and depth of Ca^2+^ signaling as its regulatory networks have evolved since early prokaryotic life. Without a doubt, learning to comprehend and communicate in a beautiful yet universal language spoken by both prokaryotic and eukaryotic lives through the intricate flow of Ca^2+^ across cellular compartments is a useful and powerful way in combating cancer and more.
